# Prenatal ultrasound diagnoses fetal amniotic band syndrome causing fetal gastroschisis: A case report

**DOI:** 10.1097/MD.0000000000046913

**Published:** 2026-01-16

**Authors:** Shiwen Xu, Xiaolin Guan, Zhihui He

**Affiliations:** a Department of Obstetrics and Gynecology, The First Affiliated Hospital of Guangzhou Medical University, Guangzhou, China.

**Keywords:** abortion, amniotic band syndrome, fetal gastroschisis, ultrasound

## Abstract

**Background:**

Amniotic band syndrome (ABS) is a congenital disease which occurs before 12 weeks of gestation. The rupture of the fiber bundle or fiber sheath around the embryo or fetus, resulting in the fetal development of this site is affected, eventually leading to deformity. But now, there are few case reports of abdominal wall fissure and visceral valgus caused by ABS. This case can broaden our horizon and let us learn more about ABS.

**Case presentation:**

Female, 25 years old, the yellow race, primiparida, color ultrasound in early pregnancy and color doppler ultrasound at 11 to 13 weeks suggest ABS. The patient and her family decided to oral mifepristone tablets and misoprostol drug to treat abortion. On the third day of drug treatment, a dead fetus was discharged from the body, and we observed that both the appearance of thoracic and abdominal wall and left foot are in accord with prenatal ultrasound diagnosis. The fetal copy number variation-seq results were“no triploid variation was detected; no chromosome aneuploid variation and known or suspected microdeletion/microduplication variation above 100 kb were detected.”

**Conclusion:**

Fetal subsequent growth and development will be seriously affected by the fetal gastroschisis, so, drug treatment is mainly treatment currently. Most of the ABS cases are sporadic cases, but also some familial cases are reported occasionally, therefore, not only can we diagnose this disease by ultrasound at 11 to 13 weeks, but also we can combine couples’ chromosome and genetic testing to exclude the related genetic variation, which can effectively prevent the occurrence of ABS familial cases.

## 1. Introduction

**Figure 1.**
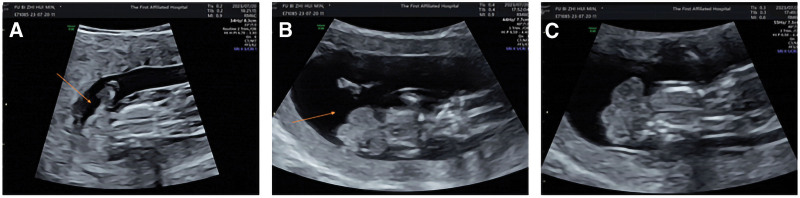
(A) The continuity of the amniotic band is interrupted, with filaments floating in the amniotic cavity, which is the fiber band produced by the amniotic membrane rupture, the amniotic fluid is less, the left foot is swollen to the extraembryonic body. (B) The continuity of the fetal abdominal wall is interrupted, and an irregular hyperechoic area (viscera) is protruding into the amniotic cavity. (C) The fetal spine is irregular.

**Figure 2.**
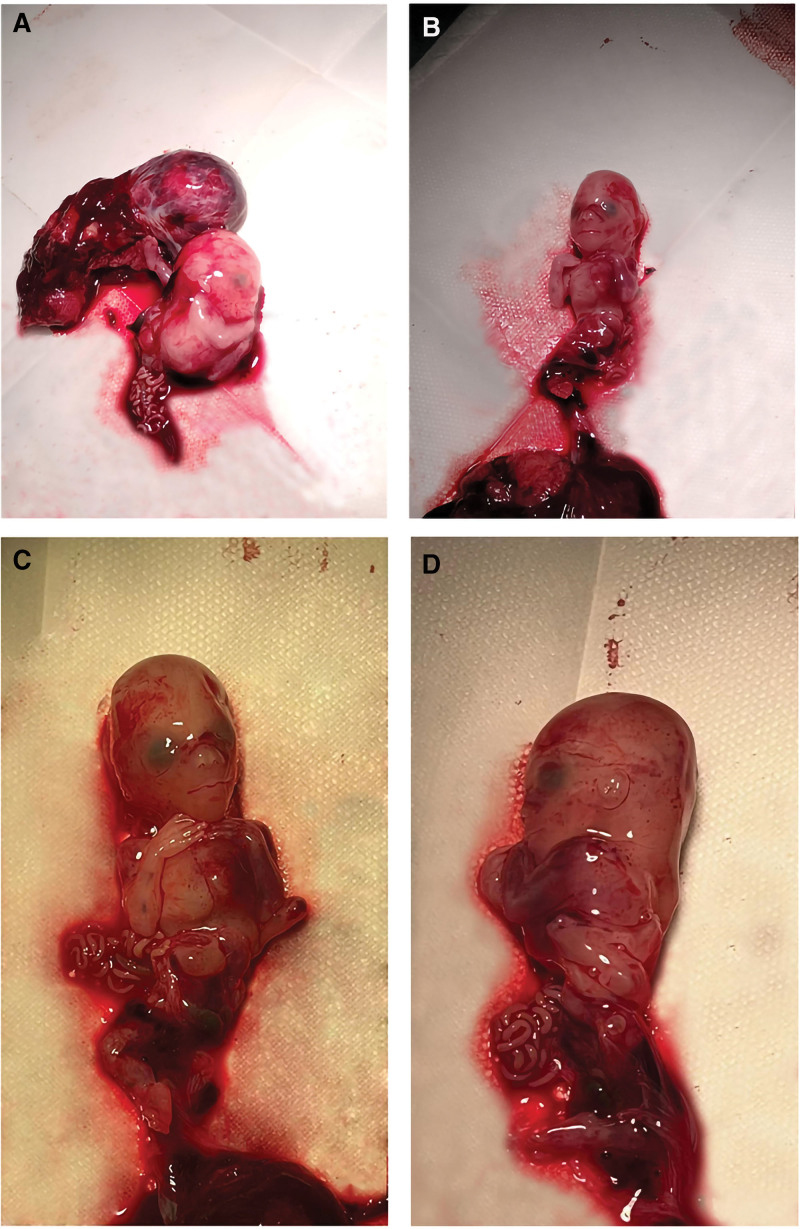
(A–D) The appearance of the thickness of the fetal neck is thick, the chest cavity is open, the size is about 0.5 × 0.5 cm, the continuity of the abdominal wall is interrupted, and an irregular mass and liver and intestinal protrusion, the size is about 2 × 1 cm, and the left lower limb is folded to the fetal back. The placental fetal membrane tissue is relatively intact, and interrupted continuity is seen in the amniotic band.

Amniotic band syndrome (ABS) is a congenital disease which occurs before 12 weeks of gestation. The rupture of the fiber bundle or fiber sheath around the embryo or fetus, resulting in the fetal development of this site is affected, eventually leading to deformity. But now, there are few case reports of fetal gastroschisis caused by ABS. This case can broaden our horizon and let us learn more about ABS (Figs. 1 and 2).

## 2. Case presentations

Female, 25 years old, chief complain: an intrauterine pregnancy 12 weeks, fetal malformation was found for 1 day. Previous regular menstruation, primiparida, without history of adverse pregnancy in the family. Color ultrasound in early pregnancy and color doppler ultrasound at 11 to 13 weeks suggest: fetal gastroschisis, nasal bone displays unclear, fetal spinal irregularity, less amniotic fluid, amniotic band continuity interruption and left foot to the external body cavity protrusion, considering ABS. It was suggested that the patient terminate the pregnancy, after considering, the patient and her family decided to oral mifepristone tablets and misoprostol drug to treat abortion. On the third day of drug treatment, a dead fetus was discharged from the body, and we observed that both the appearance of thoracic and abdominal wall and left foot are in accord with prenatal ultrasound diagnosis. The fetal copy number variation-seq results were “no triploid variation was detected; no chromosome aneuploid variation and known or suspected microdeletion/microduplication variation above 100 kb were detected.” The couple did not undergo a chromosomal examination for personal reasons. We provided the patient with psychological comfort and suggested that she should seek relevant genetic counseling before her next pregnancy. The patient and her family were satisfied with the timely treatment.

## 3. Discussion

ABS is a congenital disease with an incidence of 1/15,000 to 1/1200, which occurs before 12 weeks of gestation. The rupture of the fiber bundle or fiber sheath around the embryo or fetus, resulting in the fetal development of this site is affected, eventually leading to deformity,^[1]^ and this malformation is multisource, asymmetric, can involve all parts and organs of the fetal body, among with the limbs and trunk are the most common. The pathogenesis of ABS is not clear at present, but the theory of internal causes and external causes has become the mainstream hypothesis. The internal cause theory believes that the change of genetic material caused by the genetic factors is the main cause, such as blood vessel destruction, amniotic membrane development defects, collagen defects, coagulation defects, etc, the change of these factors leads to fetal malformation.^[2]^ The external factors, such as excessive fetal activity, swallowing or chewing the amniotic band, leading to the adhesion of the amniotic band with the fetal limb, trunk and face, thus hindering the growth of tissue in the adhesion site, which leading to fetal mechanical damage such as abdominal wall fissure, skull and facial deformity.^[3]^ A national population-based case-control study by Helenius I et al^[4]^ showed that factors such as primiparida (OR = 2.56), advanced age (OR = 1.72), use of β blockers (aOR = 24.2) in the first trimester, and progesterone (aOR = 3.79) may increase the risk of limb deformities due to ABS. This case copy number variation-seq check normal, and through the specimen, we observe visibly that the amniotic band adhere the abdominal wall fissure, considering the formation of fetal abdominal fissure and visceral valgus is associated with ABS. But now, there are few case reports of abdominal wall fissure and visceral valgus caused by ABS. Fetal subsequent growth and development will be seriously affected by the fetal gastroschisis, so, the drug treatment is mainly treatment currently. But now, fetoscopic surgery has been gradually carried out in this regard, helping many pregnant women solve this problem.^[5]^ Studies have shown that when ABS involves a small part of the fetal limbs, it does not affect the overall development of the fetus, which can solve the associated malformations through postnatal surgery.^[6]^ And McKinney J et al^[7]^reported that most of the ABS cases are sporadic cases, but also some familial cases are reported occasionally, therefore, in clinical, in addition to use prenatal ultrasound to screen fetal ABS deformity, we can combine couples’ chromosome and genetic testing to exclude the related genetic variation, which can effectively prevent the occurrence of ABS familial cases.

## 4. Conclusions

ABS is a congenital disease which occurs before 12 weeks of gestation. The pathogenesis of ABS is not clear at present, but the theory of internal causes and external causes has become the mainstream hypothesis. Fetal subsequent growth and development will be seriously affected by the fetal gastroschisis, so, the drug treatment is mainly treatment currently. Most of the ABS cases are sporadic cases, but also some familial cases are reported occasionally, therefore, not only can we diagnose this disease by ultrasound at 11 to 13 weeks, but also we can combine couples’ chromosome and genetic testing to exclude the related genetic variation, which can effectively prevent the occurrence of ABS familial cases.

## Acknowledgments

The authors are grateful to Hongwei Yang for his help with the preparation of Figure 1 in this paper.

## Author contributions

**Supervision:** Zhihui He.

**Writing – original draft:** Shiwen Xu.

**Writing – review & editing:** Xiaolin Guan, Zhihui He.

## References

[1] da Silva AJF. Amniotic band syndrome with placenta-encephalocele adhesion: an uncommon case. J Pediatr Neurosci. 2020;15:160–1. 33042254 10.4103/jpn.JPN_163_19 PMC7519744

[2] Feng D Zhou S. Amniotic band syndrome with craniofacial malformation. J Clin Ultrasound Med. 2023;25:232 + 236.

[3] Callaghan MB Hadden R King JS Lachlan K van Dijk FS Turnpenny PD. Amniotic band sequence in paternal half-siblings with vascular Ehlers-Danlos syndrome. Am J Med Genet A. 2020;182:553–6. 31833208 10.1002/ajmg.a.61449

[4] Syvänen J Raitio A Nietosvaara Y. Risk factors and prevalence of limb deficiencies associated with amniotic band sequence: a population-based case-control study. J Pediatr Orthop. 2021;41:e94–7. 32991492 10.1097/BPO.0000000000001686

[5] Ferrer-Marquez F Peiro JL Tonni G Ruano R. Fetoscopic release of amniotic bands based on the evidence-a systematic review. Prenat Diagn. 2024;44:1231–41. 39080813 10.1002/pd.6636

[6] Poeuf B Samson P Magalon G. Syndrome des brides amniotiques [Amniotic band syndrome]. Chir Main. 2008;27(Suppl 1):S136–47. French. 18948051 10.1016/j.main.2008.07.016

[7] Gandhi M Rac MWF McKinney J; Society for Maternal-Fetal Medicine. Amniotic band sequence. Am J Obstet Gynecol. 2019;221:B5–6. 10.1016/j.ajog.2019.09.020 31787161

